# Extracellular signal-regulated kinase 1/2 plays a pro-life role in experimental brain stem death via MAPK signal-interacting kinase at rostral ventrolateral medulla

**DOI:** 10.1186/1423-0127-17-17

**Published:** 2010-03-15

**Authors:** Samuel HH Chan, Enya YH Sun, Alice YW Chang

**Affiliations:** 1 Center for Translational Research in Biomedical Sciences, Chang Gung Memorial Hospital-Kaohsiung Medical Center, Kaohsiung County 83301, Taiwan

## Abstract

**Background:**

As the origin of a life-and-death signal detected from systemic arterial pressure, which sequentially increases (pro-life) and decreases (pro-death) to reflect progressive dysfunction of central cardiovascular regulation during the advancement towards brain stem death in critically ill patients, the rostral ventrolateral medulla (RVLM) is a suitable neural substrate for mechanistic delineation of this fatal phenomenon. The present study assessed the hypothesis that extracellular signal-regulated kinase 1/2 (ERK1/2), a member of the mitogen-activated protein kinases (MAPKs) that is important for cell survival and is activated specifically by MAPK kinase 1/2 (MEK1/2), plays a pro-life role in RVLM during brain stem death. We further delineated the participation of MAPK signal-interacting kinase (MNK), a novel substrate of ERK in this process.

**Methods:**

An experimental model of brain stem death that employed microinjection of the organophosphate insecticide mevinphos (Mev; 10 nmol) bilaterally into RVLM of Sprague-Dawley rats was used, in conjunction with cardiovascular, pharmacological and biochemical evaluations.

**Results:**

Results from ELISA showed that whereas the total ERK1/2 was not affected, augmented phosphorylation of ERK1/2 at Thr202 and Tyr204 in RVLM occurred preferentially during the pro-life phase of experimental brain stem death. Furthermore, pretreatment by microinjection into the bilateral RVLM of a specific ERK2 inhibitor, ERK activation inhibitor peptide II (1 nmol); a specific MEK1/2 inhibitor, U0126 (5 pmol); or a specific MNK1/2 inhibitor, CGP57380 (5 pmol) exacerbated the hypotension and blunted the augmented life-and-death signals exhibited during the pro-life phase. Those pretreatments also blocked the upregulated nitric oxide synthase I (NOS I)/protein kinase G (PKG) signaling, the pro-life cascade that sustains central cardiovascular regulatory functions during experimental brain stem death.

**Conclusions:**

Our results demonstrated that activation of MEK1/2, ERK1/2 and MNK1/2 in RVLM plays a preferential pro-life role by sustaining the central cardiovascular regulatory machinery during brain stem death via upregulation of NOS I/PKG signaling cascade in RVLM.

## Background

Although brain stem death is currently the legal definition of death in Taiwan and many countries [1,2], the detailed cellular and molecular mechanisms underlying this phenomenon of paramount medical importance are still unclear. The invariable prognosis that asystole occurs within hours or days after the diagnosis of brain stem death [3] strongly suggests that permanent impairment of the brain stem cardiovascular regulatory machinery precedes death. Better understanding of the mechanistic aspects of the dysfunction of central cardiovascular regulation during brain stem death should therefore enrich the dearth of information currently available on this fatal phenomenon.

One suitable experimental animal model for mechanistic evaluation of brain stem death uses the organophosphate poison mevinphos (3-(dimethoxyphosphinyloxyl)-2-butenoic acid methyl ester (Mev), a US Environmental Protection Agency Toxicity Category I pesticide, as the experimental insult [4]. At the same time, as the origin of a life-and-death signal [5] that reflects failure of the central cardiovascular regulatory machinery during brain stem death [6-8] and a brain stem site via which Mev acts to elicit cardiovascular toxicity [9], the rostral ventrolateral medulla (RVLM) is a suitable neural substrate for mechanistic evaluation of this fatal phenomenon [4]. Of interest is that the waxing and waning of the life-and-death signal, which mirrors the fluctuation of neuronal functionality in RVLM, presents itself as the low-frequency (LF) component in the systemic arterial pressure (SAP) spectrum of intensive-care unit patients [6-8]. More importantly, the distinct phases of augmentation followed by reduction of the LF power exhibited during Mev intoxication [10-13] can be designated the pro-life and pro-death phase of central cardiovascular regulation in this model of brain stem death [4]. Based on this model, our laboratory has previously demonstrated that nitric oxide (NO) generated by NO synthase I (NOS I) in RVLM, followed by activation of the soluble guanylyl cyclase/cyclic GMP/protein kinase G (PKG) cascade, is responsible for the pro-life phase; peroxynitrite formed by a reaction between NOS II-derived NO and superoxide anion underlies the pro-death phase [10-13].

As death represents the end of existence for an individual, we proposed previously [4] that multiple pro-life and pro-death programs must be activated in RVLM during the progression toward brain stem death. Therefore, one meaningful direction in our search for the cellular and molecular mechanisms of brain stem death is to identify these regulatory programs. In this regard, the extracellular signal-regulated kinases (ERKs) present themselves as another reasonable candidate for the pro-life program. As a member of the mitogen-activated protein kinases (MAPKs), ERK1/2 pathway is activated specifically by MAPK kinase 1/2 (MEK1/2), and is an important signal for cell survival [14-19]. Sustained inhibition of ERK expression leads to the induction of apoptosis in rat neuronal PC12 cells [15]. On the other hand, ERK is strongly and persistently activated during cell survival [16,17]. With particular relevance to this study, ERK1/2 activation leads to NOS I induction in rat PC12 cells [18] and in rat aortic smooth muscle cells [19].

Based on our Mev intoxication model [4], the present study evaluated the hypothesis that MEK1/2 and ERK1/2 in RVLM play a pro-life role during brain stem death by activating the NOS I/PKG cascade. We further delineated the participation of MAPK signal-interacting kinase (MNK), a novel substrate of ERK [20,21] in this process. Our results demonstrated that activation of MEK1/2, ERK1/2 and MNK1/2 in RVLM plays a preferential pro-life role by sustaining central cardiovascular regulatory functions during brain stem death via upregulation of the NOS I/PKG signaling cascade in RVLM.

## Methods

Adult male Sprague-Dawley rats (285-345 g, n = 164) purchased from the Experimental Animal Center of the National Science Council, Taiwan, Republic of China were used. All experimental procedures carried out in this study have been approved by the Laboratory Animal Committee of the Chang Gung Memorial Hospital-Kaohsiung Medical Center, and were in compliance with the guidelines for animal care set forth by this Committee.

### General preparation

Preparatory surgery was carried out under an induction dose of pentobarbital sodium (50 mg/kg, i.p.), and included cannulation of a femoral artery and a femoral vein, together with tracheal intubation. During the recording session, which routinely commenced 60 min after the administration of pentobarbital sodium, anesthesia was maintained by intravenous infusion of propofol (Zeneca, Macclesfield, UK) at 20-25 mg/kg/h. We have demonstrated previously [22] that this scheme provided satisfactory anesthetic maintenance while preserving the capacity of central cardiovascular regulation. Body temperature of the animals was maintained at 37°C with a heating pad, and rats were allowed to breathe spontaneously with room air during the entire recording session.

### Animal model of brain stem death

The Mev intoxication model of brain stem death [4] was used. Since Mev induces comparable cardiovascular responses on given systemically or directly to RVLM [9], we routinely microinjected Mev bilaterally into RVLM to elicit site-specific effects [9-13]. SAP signals recorded from the femoral artery were simultaneously subjected to on-line power spectral analysis [9-13,23]. We were particularly interested in the low-frequency (LF; 0.25-0.8 Hz) component in the SAP spectrum because its power density mirrors the prevalence of baroreceptor reflex (BRR)-mediated sympathetic neurogenic vasomotor discharges that emanate from this brain stem site [23]. More importantly, our laboratory demonstrated previously [10-13] that the power density of this spectral signal exhibits biphasic changes that reflect the pro-life and pro-death phases seen during the progression towards brain stem death in patients who succumbed to organophosphate poisoning [8]. Heart rate (HR) was derived instantaneously from SAP signals. Temporal changes in the power density of the LF component, pulsatile SAP, mean SAP (MSAP) and HR were routinely followed for 180 min after Mev administration in an on-line and real-time manner.

### Microinjection of test agents

Microinjection bilaterally of test agents into RVLM, each at a volume of 50 nl, was carried out stereotaxically and sequentially [9-13] via a glass micropipette connected to a 0.5-μl Hamilton (Reno, NV, USA) microsyringe. The coordinates used were: 4.5-5 mm posterior to lambda, 1.8-2.1 mm lateral to midline, and 8.1-8.4 mm below the dorsal surface of cerebellum. These coordinates were selected to cover the ventrolateral medulla at which functionally identified sympathetic premotor neurons reside [24]. Test agents used included Mev (kindly provided by Huikwang Corporation, Tainan, Taiwan), a specific ERK2 inhibitor [25], ERK activation inhibitor peptide II (Calbiochem, San Diego, CA, USA); a specific MEK1/2 inhibitor [26,27], U0126 (Calbiochem); or a specific MNK1/2 inhibitor [28,29], CGP57380 (Tocris, Ellisville, MO, USA). All test agents used for pretreatments were given 30 min before the administration of Mev. The doses were adopted from previous reports [25-29] that used those test agents for the same purpose as in this study. Application of the same amount of artificial cerebrospinal fluid (aCSF) controlled for possible volume effect of microinjection of Mev, and 0.2% DMSO serving as the vehicle control for ERK activation inhibitor peptide II, U0126 or CGP57380. The composition of aCSF was (mM): NaCl 117, NaHCO_3 _25, glucose 11, KCl 4.7, CaCl_2 _2.5, MgCl_2 _1.2 and NaH_2_PO_4 _1.2. To avoid the confounding effects of drug interactions, each animal received only one pharmacological treatment.

### Collection of tissue samples from ventrolateral medulla

We routinely collected tissue samples for subsequent biochemical evaluations [10-13] during the peak of the pro-life and pro-death phase (Mev group), or 30 or 180 min after microinjection of aCSF or 0.2% DMSO into RVLM (vehicle control group). Animals were killed with an overdose of pentobarbital sodium and tissues from both sides of the ventrolateral medulla, at the level of RVLM (0.5-1.5 mm rostral to the obex), were collected by micropunches made with a 1 mm (i.d.) stainless-steel bore to cover the anatomical boundaries of RVLM. Medullary tissues collected from anesthetized animals but without treatment served as the sham-controls. The concentration of total proteins extracted from those tissue samples was determined by the BCA protein assay (Pierce, Rockford, IL, USA).

### ELISA for ERK or phosphorylated ERK

Cell lysate from ventrolateral medulla was subjected to enzyme-linked immunosorbent assay (ELISA) according to the manufacturer's protocol of a commercial kit (Cell Signaling, Danvers, MA, USA) to detect the levels of ERK1/2 or phosphorylated ERK1/2 at Thr202/Tyr204. The final absorbance of reaction solution at 450 nm was determined by spectrophotometry using an ELISA microtiter plate reader (Anthros Labtec, Salzburg, Austria), and expressed as fold changes against sham-controls.

### Western blot analysis

Western blot analysis [10-13,23] was carried out using a rabbit polyclonal antiserum against NOS I, NOS II (Santa Cruz, Santa Cruz, CA, USA) or PKG (Calbiochem); or a mouse monoclonal antiserum against nitrotyrosine (Upstate, Lake Placid, NY, USA) or β-actin (Chemicon, Temecula, CA, USA). This was followed by incubation with horseradish peroxidase-conjugated donkey anti-rabbit IgG (Amersham Biosciences, Little Chalfont, Bucks, UK) for NOS I, NOS II or PKG, or sheep anti-mouse IgG (Amersham Biosciences) for nitrotyrosine or β-actin. Specific antibody-antigen complex was detected by an enhanced chemiluminescence Western blot detection system (NEN, Boston, MA, USA). The amount of protein product was quantified by the ImageMaster Video Documentation System (Amersham Pharmacia Biotech), and was expressed as the ratio to β-actin protein.

### Histology

In some animals that were not used for biochemical analysis, the brain stem was removed at the end of the physiological experiment and fixed in 30% sucrose in 10% formaldehyde-saline solution for at least 72 h. Frozen 25-μm sections of the medulla oblongata stained with neural red were used for histological verification of the microinjection sites.

### Statistical analysis

All values are expressed as mean ± SEM. The averaged value of MSAP or HR calculated every 20 min after administration of test agents or vehicle, the sum total of power density for the LF component in the SAP spectrum over 20 min, or the protein expression level in RVLM during each phase of experimental brain stem death, were used for statistical analysis. One-way or two-way ANOVA with repeated measures was used, as appropriate, to assess group means. This was followed by the Scheffé multiple-range test for *post hoc *assessment of individual means. *P *< 0.05 was considered to be statistically significant.

## Results

### Mev intoxication model of brain stem death

**Figure 1:**
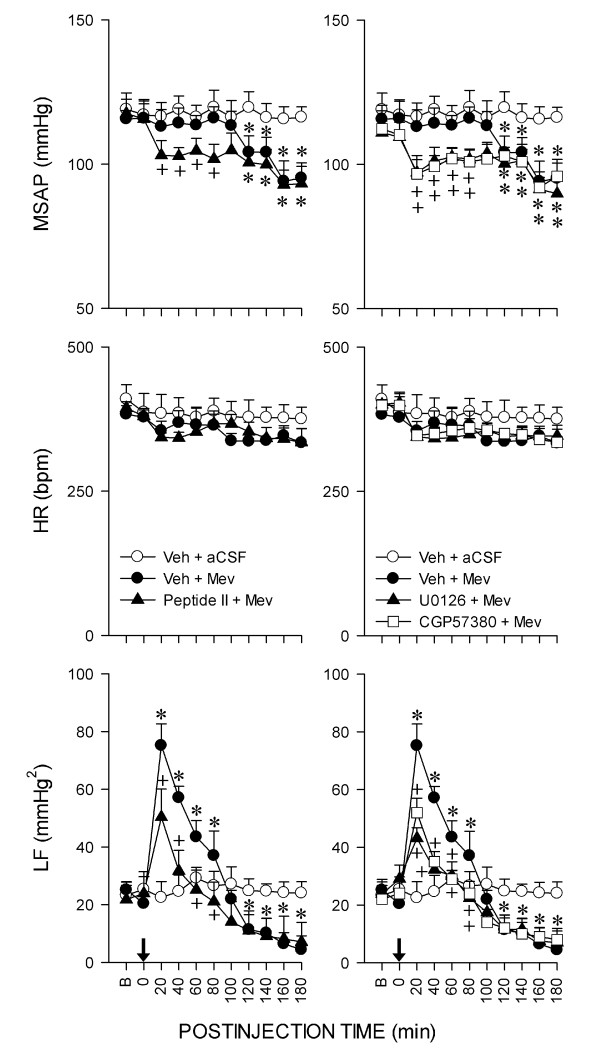
**Activation of MEK1/2, ERK1/2 or MNK1/2 in RVLM sustained central cardiovascular regulation associated with experimental brain stem death**. Temporal changes in mean systemic arterial pressure (MSAP), hear rate (HR) or power density of low-frequency (LF) component of SAP signals in rats that received pretreatment by microinjection bilaterally into RVLM of vehicle (Veh; aCSF or 0.2% DMSO), ERK activation inhibitor peptide II (Peptide II; ERK2 inhibitor), U0126 (MEK1/2 inhibitor) or CGP57380 (MNK1/2 inhibitor), 30 min before local application (at arrow) of aCSF or Mev (10 nmol) to the bilateral RVLM. Values are mean ± SEM, n = 5-7 animals per experimental group. *P < 0.05 versus Veh+aCSF group, and ^+^*P *< 0.05 versus Veh+Mev group at corresponding time-points in the Scheffé multiple-range test.

Figure 1 shows that co-microinjection bilaterally of Mev (10 nmol) and vehicle into RVLM elicited a progressive hypotension that became significant 100 min after application, accompanied by indiscernible alterations in HR. Concurrent changes in power density of the LF component of SAP signals revealed two distinct phases [10-13]. The pro-life Phase I entailed a significantly augmented LF power that endured 80-100 min to reflect sustained brain stem cardiovascular regulatory functions. The pro-death Phase II, which lasted the remainder of our 180-min observation period, exhibited further and significant reduction in the power density of this spectral component to below baseline, which signifies failure of central cardiovascular regulation that precedes brain stem death [4].

### Preferential activation of ERK1/2 in RVLM during the pro-life phase

**Figure 2:**
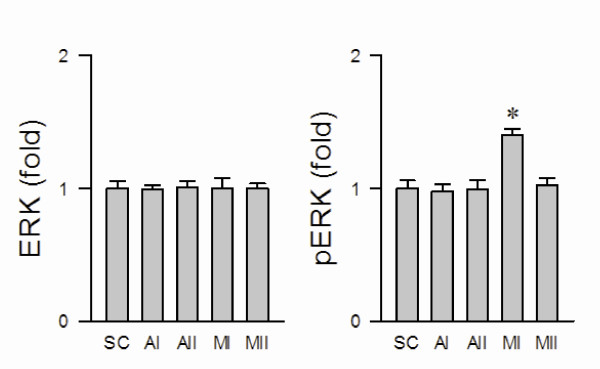
**Activation of ERK1/2 in RVLM during the pro-life phase of experimental brain stem death**. Changes in total ERK or phosphorylated ERK at Thr202 and Tyr204 in folds relative to sham-control (SC), detected in ventrolateral medulla during the pro-life phase I (MI) or pro-death phase II (MII) of experimental brain stem death or during comparable time points after treatment with aCSF (AI or AII). Values are presented as mean ± SEM of triplicate analyses on tissue samples pooled from 5-7 animals in each experimental group. *P < 0.05 versus corresponding aCSF group (AI or AII) in the Scheffé multiple-range analysis.

We first evaluated the fundamental premise that ERK1/2 in RVLM is activated during experimental brain stem death. Quantification by ELISA revealed that the total ERK1/2 in ventrolateral medulla was not affected by microinjection of Mev into the bilateral RVLM (fig. 2). Interestingly, phosphorylated ERK1/2 (pERK1/2) at Thr202 and Tyr204 in RVLM was significantly and preferentially augmented during the pro-life phase (fig. 2), of which returned to baseline during the pro-death phase. The level of both ERK1/2 and pERK1/2 in ventrolateral medulla of vehicle groups was comparable to sham-controls.

### Activation of MEK1/2, ERK1/2 or MNK1/2 in RVLM sustains central cardiovascular regulation associated with experimental brain stem death

Based on the stipulation that the magnitude and duration of the LF component of SAP signals during experimental brain stem death reflect the prevalence of the life-and-death signal [4], we next employed pharmacological blockade to evaluate whether a causal relationship exists between activation of ERK1/2 in RVLM and central cardiovascular regulation during brain stem death. Pretreatment with microinjection into the bilateral RVLM of ERK activation inhibitor peptide II (1 nmol), which binds specifically to ERK2 to prevent its interaction with MEK [25], exacerbated significantly the hypotension and blunted the augmented power density of the LF component of SAP signals during the pro-life phase (fig. 1), without affecting HR. Similar results were obtained on local application bilaterally into RVLM of U0126 (5 pmol), a specific inhibitor of MEK1 and MEK2 [26,27] (fig. 1). Those pretreatments also significantly shortened the pro-life phase to 25-35 min by shifting the prevailing phase of the 180-min observation period toward the pro-death phase (fig. 1). Intriguingly, comparable results were also obtained on pretreatment with microinjection bilaterally into RVLM of CGP57380 (5 pmol), a specific cell-permeable MNK1/2 inhibitor [28,29] (fig. 1); although a dose of 1 pmol was ineffective against the cardiovascular responses during the pro-life phase (maximal MSAP: 112.5 ± 5.2 versus 113.0 ± 5.8 mmHg; maximal HR: 356.8 ± 20.1 versus 354.6 ± 16.6 bpm; maximal LF power: 73.6 ± 6.7 versus 75.2 ± 7.5 mmHg^2 ^when compared to 0.2% DMSO pretreatment; mean ± SEM, n = 4 animals). On the other hand, ERK activation inhibitor peptide II, U0126 or CGP57380 did not significantly affect the hypotension and decrease in LF power already exhibited during the pro-death phase. Furthermore, pretreatment with vehicles exerted minimal effects on the phasic cardiovascular responses.

### Activation of MEK1/2 or ERK1/2 underlies the augmentation of NOS I or PKG in RVLM during the pro-life phase

**Figure 3:**
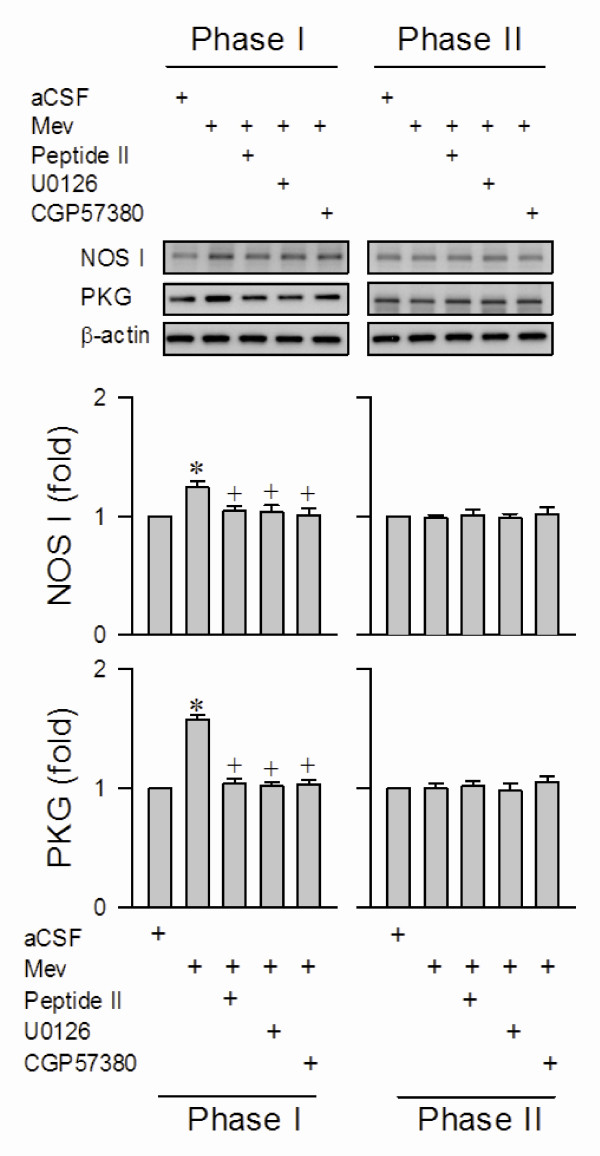
**Activation of MEK1/2, ERK1/2 or MNK1/2 leads to phasic upregulation of NOS I/PKG cascade in RVLM during experimental brain stem death**. Illustrative gels or summary of fold changes against aCSF controls in ratio of nitric oxide synthase I (NOS I) or protein kinase G (PKG) relative to β-actin protein detected in ventrolateral medulla of rats that received ERK activation inhibitor peptide II, U0126 or CGP57380 into bilateral RVLM, 30 min before induction of experimental brain stem death. Values are mean ± SEM of triplicate analyses on samples pooled from 5-7 animals per experimental group. *P < 0.05 versus aCSF group and ^+^*P *< 0.05 versus Mev group in the Scheffé multiple-range test.

We previously demonstrated [10-13] that NOS I/PKG signaling in RVLM is responsible for sustaining central cardiovascular regulation during the pro-life phase in our Mev intoxication model of brain stem death. It is therefore conceivable that MEK1/2 or ERK1/2 in RVLM may confer its pro-life actions via the NOS I/PKG cascade. As reported previously [10-13], Western blot analysis revealed a significant augmentation of NOS I or PKG expression in ventrolateral medulla during the pro-life phase, followed by a return to baseline during the pro-death phase (fig. 3). Pharmacological blockade was again used to ascertain that these temporally correlated biochemical changes are causally linked to MEK1/2 or ERK1/2 activation in RVLM during experimental brain stem death. Pretreating animals by microinjection into the bilateral RVLM of ERK activation inhibitor peptide II (1 nmol) or U0126 (5 pmol) significantly blunted the augmented NOS I or PKG protein expression at ventrolateral medulla during the pro-life phase (fig. 3). On the other hand, the protein levels of NOS I and PKG during the pro-death phase were not affected by these pretreatments.

### Activation of MEK1/2 or ERK1/2 is not responsible for the augmentation of NOS II or peroxynitrite in RVLM during the pro-death phase

**Figure 4:**
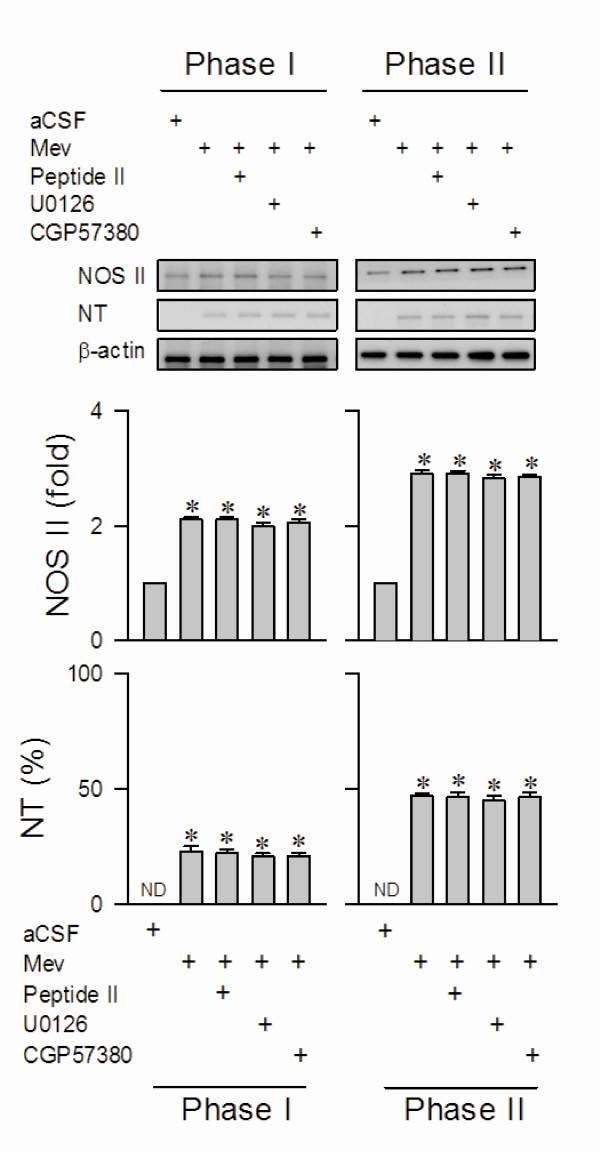
**Activation of MEK1/2, ERK1/2 or MNK1/2 did not affect NOS II/peroxynitrite signaling in RVLM during experimental brain stem death**. Illustrative gels or summary of fold changes against aCSF controls in ratio of NOS II or nitrotyrosine (NT; marker for peroxynitrite) relative to β-actin protein detected in ventrolateral medulla of rats that received ERK activation inhibitor peptide II, U0126 or CGP57380 into bilateral RVLM, 30 min before induction of brain stem death. Note that NT is presented as % relative to β-actin because it is below detection limit (ND) in aCSF controls. Values are mean ± SEM of triplicate analyses on samples pooled from 5-7 animals per experimental group. *P < 0.05 versus aCSF group and ^+^*P *< 0.05 versus Mev group in the Scheffé multiple-range test.

We also demonstrated in our previous studies [10-13] that a progressive augmentation of NOS II and nitrotyrosine (an experimental index for peroxynitrite) expression in RVLM underlies the failure of central cardiovascular regulatory functions during experimental brain stem death. As such, MEK1/2 or ERK1/2 activation in RVLM may also lead to an antagonism of this augmentation. However, pretreatment with ERK activation inhibitor peptide II (1 nmol) or U0126 (5 pmol), similar to the vehicle controls, exerted no influence against the increase of NOS II and nitrotyrosine protein expression in ventrolateral medulla during both phases of experimental brain stem death (fig. 4).

### Activation of MNK1/2 leads to upregulation of NOS I or PKG in RVLM during the pro-life phase

Our final series of experiments investigated whether activation of MNK1/2 is upstream to the augmented NOS I or PKG expression in RVLM during the pro-life phase. Western blot analysis again revealed that microinjection bilaterally into RVLM of an effective dose of CGP57380 (5 pmol) significantly blunted the elevated NOS I or PKG protein level at ventrolateral medulla during the pro-life phase of experimental brain stem death (fig. 3), without affecting the increase of NOS II or nitrotyrosine protein expression (fig. 4).

## Discussion

Based on a clinically relevant experimental model [4], the present study provided novel demonstrations that MEK1/2 or ERK1/2 activation in RVLM sustains central cardiovascular regulation during the progression towards brain stem death. We further showed that mechanistically, this pro-life role was executed via upregulation of the pro-life NOS I/PKG signaling cascade by MNK1/2.

It is generally contended that of the three MAPKs characterized in mammals, ERK1/2 plays a crucial role in survival responses [30-33]. On the other hand, Jun N-terminus kinase (JNK) and p38 MAPK primarily mediate inflammatory response [34,35] and promote cell death [36-38]. In rats that received transient middle cerebral artery occlusion, pERK is strongly expressed at the ischemic penumbra in cerebral cortex and is essentially involved in cell survival [33]. The MEK/ERK survival pathway mediates neuroprotection of striatal neurons [32] or hippocampal CA1/CA3 neurons [31] against glutamatergic neuronal cell death. It also protects sympathetic neurons against apoptosis induced by the antimitotic nucleoside cytosine arabinoside [30]. The present study further identified a novel survival function for MEK1/2 or ERK1/2 at RVLM in the form of a pro-life role during experimental brain stem death.

We previously demonstrated a pro-life role for NOS I/PKG cascade at RVLM in experimental brain stem death [10-13]. The present study further revealed that this pro-life signaling cascade is downstream to activation of MEK/ERK in RVLM. This demonstration is echoed by the observation [19] that gene transfer or gene knockdown of MEK increases or decreases NOS I expression in cultured rat aortic smooth muscle cells. Moreover, the MEK/ERK pathway, but not JNK or p38 MAPK, is required for NOS I mRNA or protein expression and activity in PC12 cells [18]. There are two possible, though not necessarily mutually exclusive, mechanisms for MEK/ERK to elicit NOS I induction. One possibility is for ERK1/2 to transcriptionally upregulate NOS I. The promoter region of NOS I gene contains putative cis-elements of binding sites for cAMP response element (CREB), C/EBP and c-Myc [39,40], which are candidates for ERK nuclear targeting in the mediation of gene transcription. Another possibility is for ERK to exert posttranslational modification by phosphorylation of NOS I protein. ERK1/2 is tightly bound to its physiologically relevant substrates, such as MNKs and p90RSK1 for subsequent physiological responses. Whereas p90RSK1 is a well-known substrate for ERK1/2, MNK1 and MNK2 are novel serine/threonine protein kinases that could be phosphorylated by ERK1/2 [20]. MNK1 activation is inhibited by MEK inhibitor PD98059 [20,21], suggesting that it is an important regulator for MNK activation. It follows that MNK1/2 may enhance phosphorylation of NOS I on activation by MEK/ERK. Whether the implied augmentation of NOS I or PKG expression in RVLM by MNK1/2 observed during experimental brain stem death in the present study entails transcriptional upregulation remains to be investigated.

Our results also showed that the pro-life role of MEK, ERK and MNK in RVLM during experimental brain stem death is manifested by sustaining central cardiovascular regulation. In this regard, it is of interest to note that these cellular signals may be linked to angiotensin II (Ang II), a well-known peptide that is crucial to the elevation of SAP in RVLM. Activation of the ERK/CREB/c-fos cascade mediates the long-term pressor effect of Ang II in RVLM [26]. MEK and ERK1/2 also participate in Ang II-induced vascular smooth muscle cell contraction [41]. Furthermore, MNK mediates Ang II-induced protein synthesis in vascular smooth muscle cells [29]. Whether Ang II in RVLM plays a pro-life role during brain stem death via activation of the MEK/ERK/MNK cascade, however, awaits documentation.

## Conclusion

In conclusion, the present study revealed that the MEK/ERK/MNK cascade in RVLM plays a pro-life role during experimental brain stem death by sustaining the central cardiovascular regulatory machinery via NOS I/PKG signaling.

## Competing interests

The authors declare that they have no competing interests.

## Authors' contributions

EYHS performed the physiological experiments and carried out the ELISA. SHHC and AYWC conceived the study, participated in experimental design, and drafted and revised the manuscript. All authors have read and approved the final manuscript.
